# Margin-Based Deep Learning Networks for Human Activity Recognition

**DOI:** 10.3390/s20071871

**Published:** 2020-03-27

**Authors:** Tianqi Lv, Xiaojuan Wang, Lei Jin, Yabo Xiao, Mei Song

**Affiliations:** School of Electronic Engineering, Beijing University of Posts and Telecommunications, Beijing 100876, China; lvtianqi@bupt.edu.cn (T.L.); jinlei@bupt.edu.cn (L.J.); xiaoyabo@bupt.edu.cn (Y.X.); songm@bupt.edu.cn (M.S.)

**Keywords:** human activity recognition, deep learning, margin mechanism, open-set classification

## Abstract

Human activity recognition (HAR) is a popular and challenging research topic, driven by a variety of applications. More recently, with significant progress in the development of deep learning networks for classification tasks, many researchers have made use of such models to recognise human activities in a sensor-based manner, which have achieved good performance. However, sensor-based HAR still faces challenges; in particular, recognising similar activities that only have a different sequentiality and similarly classifying activities with large inter-personal variability. This means that some human activities have large intra-class scatter and small inter-class separation. To deal with this problem, we introduce a margin mechanism to enhance the discriminative power of deep learning networks. We modified four kinds of common neural networks with our margin mechanism to test the effectiveness of our proposed method. The experimental results demonstrate that the margin-based models outperform the unmodified models on the OPPORTUNITY, UniMiB-SHAR, and PAMAP2 datasets. We also extend our research to the problem of open-set human activity recognition and evaluate the proposed method’s performance in recognising new human activities.

## 1. Introduction

Human activity recognition (HAR), the goal of which is to identify specific activities carried out by a person (or persons), has gained much attention recently due to its wide range of applications in many fields, such as healthcare [1], athletic competitions [2], and smart cities [3]. HAR methods can be divided as two categories: vision-based and sensor-based. Due to the complex backgrounds of images and strict demands of environmental conditions, the actual application range of vision-based HAR remains limited. Sensor-based methods are more robust in variable environments and have high recognition accuracy and low power consumption, allowing these methods to be more widely used.

In traditional sensor-based HAR methods, hand-crafted features are crafted such that the classifier can recognise different activities. The drawback of these methods is that they rely heavily on human experience or domain knowledge. In recent years, with the rapid development of deep learning technology, the classification performance of HAR based on deep learning networks has increased substantially [4,5]. Compared with traditional methods, deep learning networks can automatically extract high-dimensional features from raw sensor inputs. Although recent studies have achieved good classification performance [6,7,8], current HAR systems are still far from ideal. The first issue which we deal with in this work is that some human activities have intra-class diversity and inter-class similarity. Intra-class diversity occurs when the same activities have large variability when carried out by different people. Inter-class similarity means that different activities are highly similar, such as the "Open Door" and "Close Door" classes in the OPPORTUNITY dataset. Meanwhile, some researchers have also found that softmax loss does not acquire discriminative features [9,10,11]. The second problem which often appears in classification tasks is that, in practice, new activities are likely to appear in an HAR system. This means that HAR can be regarded as an open-set classification problem. Open-set HAR is essentially a metric learning problem, where the effective solution obtains discriminative large-margin features. In view of these challenges, we incorporate a margin mechanism with softmax loss to improve the discriminative power of deep learning networks. We use the deep learning models proposed by [5] to prove the effectiveness of our method on three open human activity datasets: OPPORTUNITY [12], UniMiB-SHAR [13], and PAMAP2 [14]. We also design a framework to deal with open-set HAR and evaluate the effectiveness of this framework on the PAMAP2 dataset. Our contributions are as follows:

- We add a margin mechanism to the softmax loss to learn more discriminative feature representations which enhance intra-class compactness and inter-class diversity.
- We use four deep learning models to carry out comparison experiments on three widely used public datasets and prove that the margin-based method can improve the recognition ability of the deep learning networks. Furthermore, we also conduct experiments to compare three machine learning classifiers trained using hand-crafted features and features from the unmodified and margin-based deep learning networks.
- We illustrate how the margin-based networks outperformed the unmodified models with different hyperparameters. Additionally, we carry out experiments to visualise the cosine similarity and 2-D learning features of the softmax and additive angular margin losses.
- We extend our study to the open-set HAR problem. To the best of our knowledge, our work is the first to treat HAR as an open-set classification problem. We confirm the effectiveness and performance of our method using the PAMAP2 dataset.

The rest of this paper is organised as follows: Section 2 provides a brief overview of related works on HAR, including traditional methods for HAR, deep learning for HAR, and metric learning. In Section 3, we propose our selection of deep learning models and margin-based loss, as well as explaining our method for dealing with the open-set HAR problem. We introduce three benchmark datasets, the performance metrics, and experimental settings in Section 4. Section 5 provides evaluation results, in terms of the three datasets, and the open-set HAR results. We present our conclusions in Section 6.

## 2. Related Work

According to the different processes of feature extraction in HAR based on wearable sensor data, the related works can be mainly summarised as belonging to two categories: hand-crafted feature-based traditional methods and auto-extracted feature-based deep learning methods. Traditional methods attempt to completely describe the input data with some machine learning models, such as Support Vector Machines (SVM), Naive Bayes (NB), k-nearest Neighbours (KNN), Random forest (RF), and so on. Chen et al. [15] proposed an online-independent support vector machine (OISVM) to update the parameters of SVM online based on a small portion of data and experimentally demonstrated the effectiveness of the OISVM algorithm. Hossain et al. [16] compared the recognition performance of NB and RF based on some statistical features in the presence of different levels of random missing data. The results showed that NB provides better performance than RF in both simulated and real data of the HASC dataset. Mobark et al. [17] collected data about breakfast scenarios and divided them into three levels, according to their complexity. They carried out experiments in seven classifiers and the best results were achieved by the KNN classifier. Xu et al. [6] proposed a novel human activity recognition method based on RF. Their method achieved an overall accuracy of about 90% on wearable device data.

In contrast to machine learning methods with shallow statistical features, deep learning methods can extract deep features automatically and have achieved superior performance in HAR. The deep learning networks used in HAR tasks include Convolutional Neural Networks (CNNs), Recurrent Neural Networks (RNNs), Multi-Layer Perceptrons (MLPs), Autoencoders (AEs), and so on. Panwar et al. [7] designed a CNN model for three activities using a single wrist-worn accelerometer sensor. Their experiments showed that the CNN model outperforms SVM, K-means, and LDA. Huang et al. [8] proposed a two-stage end-to-end CNN network and a data augmentation method. Their proposed method achieved significant recognition accuracy, compared with the state-of-the-art methods, as well as reduced computational complexity. Vepakomma et al. [18] extracted hand-crafted features from sensor data and fed them into a MLP model. This model could recognise 22 complex daily actions with high average test accuracy (90%). Almaslukh et al. [19] designed a stacked AE and first adopted the greedy layer-wise pre-training technique. RNNs, which are good at capturing intrinsic time dependencies of data, are popular in speech recognition and natural language processing. However, they suffer from the vanishing gradient problem; a variety of RNN, called Long Short-Term Memory (LSTM), has been proposed to deal with this problem. Many works have used LSTM for HAR tasks. For example, Yu et al. [20] designed a deep LSTM network with residual connections, which achieved an F1-score of 90.8% on the OPPORTUNITY dataset. A hybrid network is a combination of deep models, which can integrate the advantages of different networks. Ordóñez and Roggen [4] designed a hybrid network based on CNN and LSTM layers. The accuracy of recognition on two datasets achieved by this hybrid architecture was higher than other models by as much as 9%.

Margin mechanisms belong to the field of metric learning. Metric learning aims to learn a similarity (distance) function which maps image pixels to embedding feature vectors modelling the similarity between images [11]. Metric learning forces deep learning models to be more discriminative and has been successfully applied to many tasks, including face recognition [21,22], person re-identification [23,24], and visual searching [25,26]. Liu et al. [11] designed a large margin softmax by adding angular constraints to each identity, in order to learn discriminative face features. However, the loss function needed many approximations, which led to uneven network training. CosFace [21] modified the softmax loss by adding a cosine margin penalty, outperforming SphereFace and greatly simplifying the implementation. Deng et al. [22] proposed an Additive Angular Margin Loss (ArcFace) to improve feature discrimination. Their experiments showed that ArcFace outperforms the state-of-the-art and is easily implemented with negligible computational overhead. Wojke N and Bewley [23] applied deep cosine metric learning to learn a feature space and confirm its effectiveness on two large-scale pedestrian re-identification datasets.

Several studies have focused on the issues of intra-class diversity and inter-class similarity [27,28]. Younes et al. [27] proposed a novel classifier, called the "Classifier for Activities with Variations" (CAV), to deal with complex activities that could be performed in a wide variety of ways. The effectiveness of CAV was illustrated on eight complex activities collected by a Qualisys video motion capture system. Kim et al. [28] proposed an ensemble method using hidden Markov models to deal with the difficulty in classifying some activities due to their intra-class variations and inter-class similarities. The proposed model achieved about 83.51% accuracy on the UCI Human Activity Recognition dataset. These two methods classify activities depending on template matching with cluster centers. When the number of activities increases, the complexity of the HAR system also increases. Our proposed method does not depend on precise data acquisition equipment or cluster methods and can be widely applied to a variety of classification models which use softmax loss, in order to increase the discriminative power of the models.

## 3. Framework

**Figure 1:**
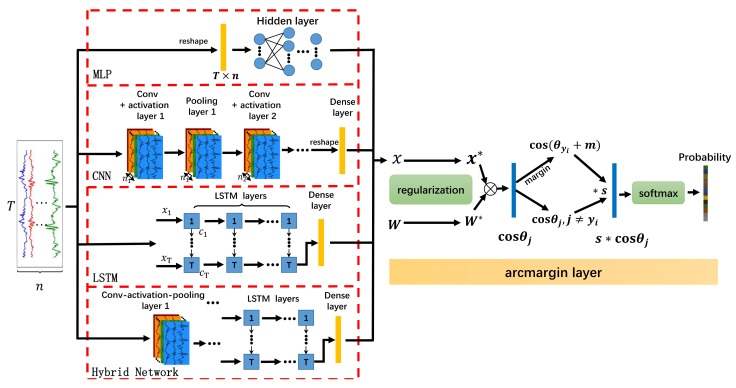
Architecture of margin-based deep learning networks.

First, we briefly describe the four deep learning models we employed. Then, we introduce the margin mechanism with which we modify the softmax function, the result of which is called the Margin-based Loss function. The overall architecture is depicted in Figure 1. Finally, the framework designed to deal with open-set HAR is detailed in Section 3.3.

### 3.1. Deep Learning Models

A MLP is the simplest artificial neural network. It includes at least three dense layers: an input layer, one or more hidden layers, and an output layer. The output of each dense layer is sent as the input to the next layer. A softmax or an arcmargin layer, which includes a fully-connected layer followed by a softmax or margin-based loss, respectively, serves as the output layer and provides predictions (in terms of probabilities) for the input data.

A CNN consists of an input layer, at least one convolutional layer followed by activity (non-linear) and pooling layers, and at least one fully-connected layer. For the convolutional layer, a local domain in a previous layer is connected to a convolution kernel to automatically learn local and short-term features. The convolution operation functions like a feature extractor coupled with an activity layer. The pooling layer applies a function (e.g., max-pooling or average-pooling) to perform downsampling, which produces translational invariance. The feature maps must be flattened before being passed through one or more of the fully-connected layers. Finally, a softmax layer computes the probability of each class.

A LSTM is used to exploit temporal dependencies within data. The design of LSTM employs gating to describe the temporal correlation between instantaneous and historical information. The LSTM model is similar to the architecture of a CNN: Input data is passed to stacked LSTM layers, and the output of the last LSTM layer is sent to a fully-connected layer. A softmax layer generates the classification probability.

A Hybrid Convolutional and Recurrent Network extracts both short- and long-term time-dependent features in the data. A hybrid model typically contains several convolutional blocks. The output of the last convolutional layer is sliced along the time dimension, with each slice subsequently flattened. The flattened vector is used as the input to the LSTM layer.

### 3.2. Margin-based Loss Function

Softmax loss has been widely used for classification problems. It aims to separate features of different classes by maximising the predicted probability of the ground-truth class. The Equation for this loss is

(1) $$L_{softmax}=-\frac{1}{m}\sum_{i=1}^{m}\log\frac{e^{f_{y_{i}}}}{\sum_{j=1}^{n}e^{f_{y_{j}}}}=-\frac{1}{m}\sum_{i=1}^{m}\log\frac{e^{W_{y_{i}}^{T}x_{i}+b_{y_{i}}}}{\sum_{j=1}^{n}e^{W_{y_{j}}^{T}x_{i}+b_{j}}},$$

where $W_{y_{i}}$ is the weight matrix of class $y_{i}$, $x_{i}$ are the feature maps, and $f_{y_{j}}$ denotes the output of a fully-connected layer with weight matrix $W_{y_{j}}$ and bias $b_{j}$. We can express $f_{y_{j}}$ as the product of norms and intersection angles, setting the $b_{j}$ to 0 for simplicity. The Equation is

(2) $$f_{y_{j}}=W_{j}^{T}x=∥W_{j}^{T}∥∥x∥\cos\theta_{j},$$

where $\theta_{j}$ is the angle between class $W_{y_{j}}$ and *x*. The Equation shows that the predicted probability benefits from both the norm and angle of vectors. To simplify our analysis, we apply L2 regularisation to fix $∥W_{y_{j}}^{*}∥=1$ and $∥x^{*}∥=1$. To rescale the product of these two normalised vectors, we multiply *s* by it. We can formulate this as

(3) $$∥W_{j}^{T}∥∥x∥=s∥{W_{j}^{*}}^{T}∥∥x^{*}∥.$$

The normalisation operation makes the predicted probability merely rely on the cosine of the angle. The following Equation is the modified loss function:

(4) $$L=-\frac{1}{m}\sum_{i=1}^{m}\log\frac{e^{s(\cos\theta_{y_{i}})}}{e^{s(\cos\theta_{y_{i}})}+\sum_{j=1,j\neq y_{i}}^{n}e^{s(\cos\theta_{j})}}.$$

Therefore, the model learns separable features in the angular space. However, the modified softmax loss can not extract discriminative features, as the loss only emphasises correct predictions. To deal with this problem, we introduce margin-based loss [22], which adds an angle interval *m* for the ground-truth class into the preceding Equation. We express this as

(5) $$L_{ArcFace}=-\frac{1}{m}\sum_{i=1}^{m}\log\frac{e^{s(\cos\left(\theta_{y_{i}}+m\right))}}{e^{s(\cos\left(\theta_{y_{i}}+m\right))}+\sum_{j=1,j\neq y_{i}}^{n}e^{s(\cos\theta_{j})}}.$$

Compared with softmax loss, the optimisation target changes from $\cos\left(\theta_{y_{i}}\right)$ to $\cos\left(\theta_{y_{i}}+m\right)$. If the network wishes to obtain the same predicted probability for a target class, the angle θ should be made smaller. This operation enhances intra-class consistency and inter-class diversity. The margin-based method is illustrated in Figure 9. The margin-based loss function is embedded in an arcmargin layer, which is shown in Figure 1.

### 3.3. Open-set Classification Problem for HAR

A closed-set classification problem requires all categories to be predefined for the training and testing sets. It is natural to classify a testing dataset for such given categories. In practice, however, all categories existing in the testing dataset may not appear in the training set, which makes the problem more challenging. For HAR, it is possible to add a new activity in an online HAR system. The features extracted using the softmax loss are not separable enough to manage open-set HAR and, so, we introduce the Additive Angular Margin Loss to learn discriminative large-margin features.

**Figure 2:**
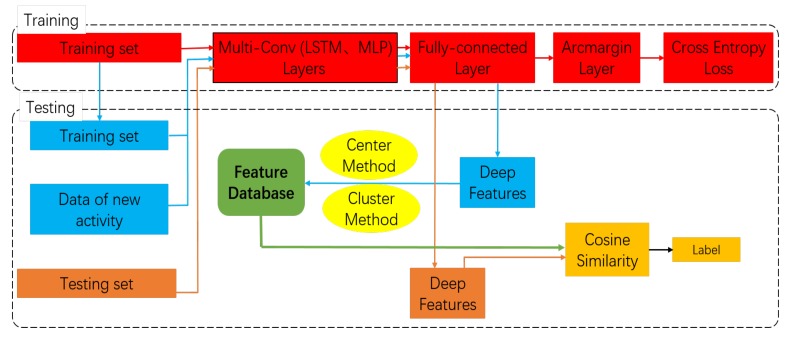
Framework for open-set human activity recognition.

We now present our proposed framework in terms of training and testing processes for open-set HAR, as shown in Figure 2. In the training stage, we use a training set with predefined classes to train the margin-based deep learning models described in Section 3. The models learn the discriminative features after training. We remove the arcmargin layer from the models and then extract high-dimensional features of the training data from the first fully-connected layer for subsequent comparison.

In order to build the feature database, representative features must be extracted from each class feature set. We propose two methods for acquiring these features: The first is the center method, which calculates the cosine similarity of each feature in a set to the other features in the same set. We assign the one with the average highest similarity as the center-seed feature and randomly select several other features as random-seed features. The second method is the cluster method. In this method, we use K-Means++ [29] to select several cluster centers as representative features for each class, which we call cluster-seed features. We construct the feature database by performing one of these two processes on the training dataset. For every class, we set a threshold, which is used to accept or refuse the prediction label of data as belonging to the class. For each predefined class, we calculate the cosine similarity between the feature of class data and the chosen seeds in the validation set. Then, we choose [0.1−0.9] (in increments of 0.1) as candidate values for the threshold. The best value of the threshold for each class is decided according to highest F1-score. For a new human activity, we calculate the cosine similarity of each sample and the other samples. We set the first quantile of these cosine similarities as the value of the new threshold (the first quantile was chosen due to it achieving the best results experimentally).

**Figure 3:**
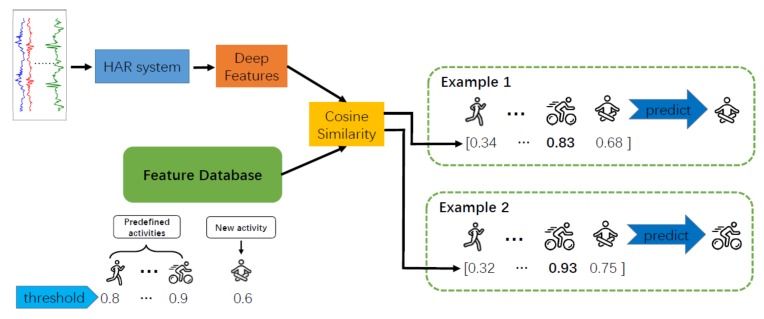
Examples of recognising an activity in open-set human activity recognition.

If a new human activity (i.e., one whose class cannot be found in the training set) appears in the HAR system, we first collect several samples and send them to the model to obtain deep features. After this, feature representations are chosen using the preceding two methods and added to the feature database to recognise the new activity. Then, the classes of the feature database include the classes in the training set and the new human activity. When it receives new data, the system obtains the deep features of the data using the model and calculates the cosine similarity between the features and the feature database. As shown in Example 1 of Figure 3, if the corresponding class for the maximum of these similarities belongs to classes in the training set and the max value is smaller than the threshold of this class but larger than the threshold of the new activity, the predicted labels of the new data form the new activity. In other cases, such as Example 2 of Figure 3, the predictions are determined by the maximum similarity.

## 4. Experiment

We used three benchmark datasets—OPPORTUNITY, UniMiB-SHAR, and PAMAP2—to prove the effectiveness of the proposed method. In this section, we first introduce the three experimental datasets. Depending on the existence of class imbalances, we further adopt two performance metrics to eliminate the influence of such imbalances. Finally, we present the parameters of four deep learning models, along with the parameters used in the training stage.

### 4.1. Benchmark Datasets

Human activities, whose features are always unique and cyclical, contain various gestures such as running, walking, sitting, and so on. Therefore, a benchmark dataset should include a variety of human activity types. Researchers have created several datasets for HAR, such as the OPPORTUNITY [12], UniMiB-SHAR [13], PAMAP2 [14], and Skoda [30] datasets. We used three datasets for the closed-set HAR problem and one for the open-set HAR problem.

The OPPORTUNITY dataset consists of four subjects performing 17 different activities in a kitchen scenario. The data was obtained using seven wireless body-worn inertial measurement units (IMUs) and 12 additional accelerometers at a sampling frequency of 30 Hz. Each subject performed six rounds: in the first five rounds, they performed scripted activities; in the last round, they repeated each activity 20 times. The data were stored in 5 ADL files and 1 Drill file. To deal with the problem of missing data, we removed 38 sensor channels, including accelerometer recordings from the left and right hands along with all quaternion channels acquired from the IMU. We used the remaining 107-dimensional data for our experiments, filling missing values from the last non-missing data.We chose the ADL1–3 and Drill files of all four subjects as the training set and the ADL4–5 files as the testing set. For frame-by-frame analysis, the length of the sliding window was 2 s and the sliding stride was 3. The resulting training and testing sets included about 211,000 and 78,000 frames, respectively.

The UniMiB-SHAR dataset was built from the recordings of a Samsung Galaxy Nexus I9250 smartphone with an embedded 3D-accelerometer. A total of 30 volunteers performed 17 activities wearing the smartphone in their left or right pocket. The data was sampled at a constant sampling rate of 50 Hz. Following previous work, we used a energy-based segmentation technique with a fixed-width sliding window of 151 (about 3 s) to slice the data [13]. The dataset consisted of approximately 11,000 frames. We carried out 30-fold leave-one-subject-out cross validation for experiments with the UniMiB-SHAR Dataset.

The PAMAP2 dataset consists of data from nine subjects (eight male and one female) arranged to perform 18 activities, including a protocol of 12 activities (e.g., lying down, cycling, and jumping rope) and six optional activities (e.g., computer work and playing soccer). Over 10 h of data were collected by IMUs worn on the hand, ankle, and chest. The resulting dataset has 52 dimensions. We chose runs 1 and 2 for subject 5 as the validation set and runs 1 and 2 for subject 6 as the test set. The remaining data was used as our training set. To obtain a temporal resolution similar to the OPPORTUNITY dataset, we downsampled the sensor data to 33.3 Hz and sliced it using sliding windows of 5.12 s with 78% overlap between adjacent windows. This yielded approximately 14,000, 2000, and 2000 frames for the training, validation, and testing sets, respectively.

### 4.2. Performance Metrics

Human activity datasets are often highly unbalanced. The NULL class of the OPPORTUNITY dataset represents more than 75% of the total data, which leads to an extremely imbalanced data distribution. For this dataset, the overall classification accuracy is not a suitable metric for measuring performance, as a classifier that predicts as many instances as possible as belonging to the majority class could have a high performance. Instead, we used the weighted F1-score to assess the models, which simultaneously considers precision and recall for each activity. Precision and recall are defined as $\frac{TP}{TP+FP}$ and $\frac{TP}{TP+FN}$, respectively, where TP, FP, and FN represent the number of true positives, false positives, and false negatives. To deal with data imbalance, the weighted F1-score calculates the F1-score of each class and then multiplies it by a weight value:

(6) $$F_{w}=\sum_{c}2*w_{c}\frac{precision_{c}\cdot recall_{c}}{precision_{c}+recall_{c}},$$

where *c* is the class index and $w_{c}=n_{c}/N$ represents the proportion of samples of the $c^{\mathrm{th}}$ class. We also evaluated two other metrics for comparison, as used by Li [5]: the overall accuracy and the average F1-score. The average F1-score is independent of the class distribution and defined as

(7) $$F_{m}=\frac{2}{\left|c\right|}\sum_{c}\frac{precision_{c}\cdot recall_{c}}{precision_{c}+recall_{c}}.$$

### 4.3. Model Settings

Table 1 shows the settings of the deep learning models introduced in Section 3. These are the same as [5], for the sake of fair comparison. For the closed-set HAR problem, the hyper-parameters of these models for the OPPORTUNITY and PAMAP2 datasets were kept consistent, but differed from those used with the UniMiB-SHAR dataset.

For all three datasets, we placed a batch normalisation layer [31] after the input of each model, in order to avoid internal covariate shift, as it demonstrated the best performance [5]. The fully-connected layers shown in Table 1 were followed by a REctified Linear Unit (RELU) activation layer to provide non-linear expression. Each of the CNN blocks in Table 1 included convolutional, RELU, and max-pooling layers. The number of cells in the LSTM layers were determined by the size of the sliding window. The LSTM cells used a sigmoid function for gate activation and a hyperbolic tangent for other activations. We placed either a softmax layer or an arcmargin layer after the last fully-connected layer to provide predictions for each class.

### 4.4. Model Training

Our deep-learning models were implemented using the PyTorch [32] library. The computing platform was equipped with an Intel E5-2620 at 2.10 GHz, 9.6 GB RAM, and an 11 GB NVIDIA 1080 Ti GPU. All parameters of the models were randomly orthogonally initialised and updated using the ADADELTA optimiser with default patameters (i.e., initial learning rate of 1) for 50 epochs. The batch size was set to 100.

## 5. Results

### 5.1. Performance Comparison

Table 2 presents a summary of the performance results of the deep learning and margin-based models, in terms of the accuracy (acc), weighted F1-score ($F_{w}$), and average F1-score ($F_{m}$). In terms of overall performance, the margin-based methods achieved the highest scores on all three datasets. Specifically, LSTM-M obtained the highest accuracy and weighted F1-scores (92.30% and 91.99%, respectively), while the best average F1-score of 71.08% was obtained by Hybrid-M on the OPPORTUNITY dataset. The NULL class of this dataset is almost 70%, while other activities are rarely more than 2%. This problem led to inadequate training in all tested models. However, matters improved for the UniMiB-SHAR and PAMAP2 datasets. The accuracy, weighted F1-score, and average F1-score of Hybrid-M increased to 77.88%, 77.29%, and 65.31%, respectively, on the UniMiB-SHAR dataset. Meanwhile, we note that the CBS method ranked second, with the margin-based methods markedly improving performance compared with the corresponding non-margin deep learning models. For the four deep learning models, our proposed methods improved performance by about 2% on average for accuracy and weighted F1-score and about 10% on average for F1-score on the PAMAP2 dataset. Taken as a whole, each deep network using a margin-based method demonstrated a visible performance improvement.

**Figure 4:**
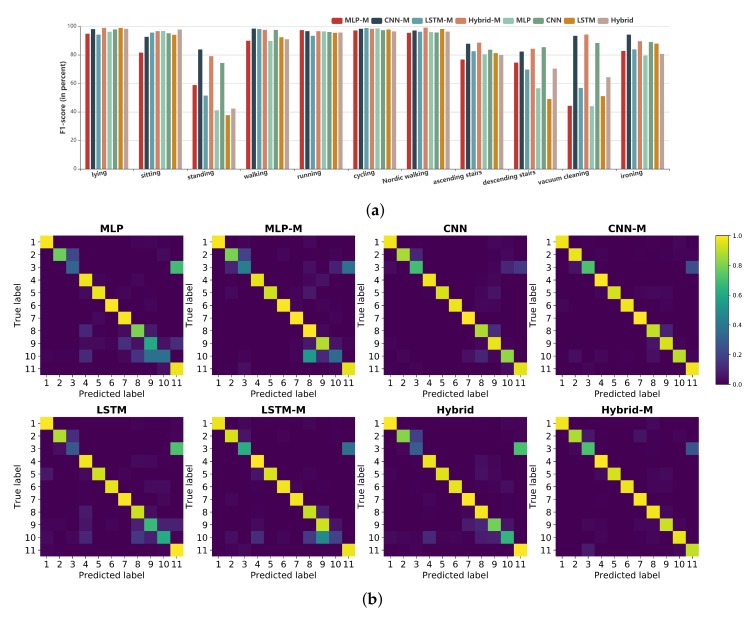
(**a**) The F1-score (in percent) of each class of different models on the PAMAP2 dataset. (**b**) Confusion matrix of each model on the PAMAP2 dataset. The horizontal and vertical axes represent the predicted and true classes, respectively. 1, lying; 2, sitting; 3, standing; 4, walking; 5, running; 6, cycling; 7, Nordic walking; 8, ascending stairs; 9, descending stairs; 10, vacuum cleaning; 11, ironing.

In order to show the effectiveness of our proposed margin-based methods more intuitively, we compared each class of the benchmark dataset between the different models. We selected only the PAMAP2 dataset for this experiment, as we trained all models reported by Li [5] using this dataset and directly used the reported performance of these models with the other two datasets. Without loss of fairness and generality, we chose the F1-score of each class as a metric. Figure 4a shows the F1-scores of each class for the deep learning models and our proposed models. Although the F1-scores of the margin-based models were very close to other models for some activities, they still performed better in all classes overall. Most notably, the highest F1-scores achieved by the Hybrid-M model were 79.08% and 94.26% for standing and vacuum cleaning, respectively; in contrast, the Hybrid model achieved only 42.31% and 64.29%, respectively. These results shows strong support for the effectiveness of our proposed methods. Our models also worked well for the minority classes of the PAMAP2 dataset. The descending stairs class accounted for only 5.7% of the total dataset, but the LSTM-M model attained an F1-score of 69.66%—an increase of 20.61%, compared with the plain LSTM model.

Figure 4b shows the confusion matrices of all networks, from which we can see that the margin-based models obtained consistently better performance than the deep learning models and had better generalisation ability for recognising complex human activities. These results might be because the learned discriminative large-margin features powerfully enhanced intra-class compactness and inter-class diversity.

Furthermore, in Table 3, we display the performance metrics of three other classifiers and the LSTM-M network on the OPPORTUNITY dataset. LSTM-M achieved excellent performance on the OPPORTUNITY dataset, as shown in Table 2. We used 18 hand-crafted features, which were computed on each sensor channel independently, according to the suggestion in [5]. From the results, we can see that the deep learning-based feature extraction method could achieves better performance, compared with the hand-crafted feature extraction methods. Furthermore, we also extracted features from the first fully-connected layer of the LSTM and LSTM-M networks, and trained the three machine learning classifiers with these features. There was a significant performance improvement for the three classifiers, especially Naïve Bayes. Classifiers which used features from the LSTM-M network outperformed the same classifier using features from the LSTM network, which also illustrates that the margin mechanism can enhance the discriminative power of deep learning methods.

### 5.2. Evaluation of Hyperparameters

#### 5.2.1. Length of Sliding Window

The length of the sliding window has a large influence on the performance of deep learning models. Human activity features are often various and cyclic. When the length of the sliding window is short, models can not extract enough features if the duration of an activity is long. However, when the length of the sliding window is too long, it contains a lot of redundant information. Therefore, we investigated the influence of different sliding window lengths on our proposed method. Besides T= 64, we conducted experiments with data sequence segments of duration T= 32 (approximately 1 s) and T= 96 (approximately 3 s).

**Figure 5:**
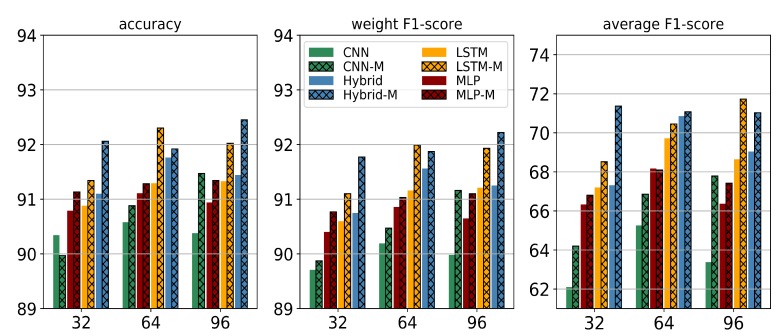
Classification performance results (in percent) using different sliding window lengths on the OPPORTUNITY dataset.

Table 4 and Figure 5 illustrate the performance of the three metrics of the unmodified and margin-based models with different sliding window lengths on the OPPORTUNITY dataset. Viewed as a whole, the margin-based method can inspire the potential of deep learning models without being affected by the various window lengths. It can also be noted that the result became better with an increase of length. This phenomenon reveals that the margin-based models could extract more discriminative features, as bigger frames probably contain more useful and redundant information. Furthermore, we also found that performances of the LSTM and Hybrid models were enhanced by the margin-based method, which proves that the proposed margin-based loss helps LSTM-based models to obtain more temporal dependencies.

#### 5.2.2. Number of Sensor Channels

Human activity recognition can use multi-modal data but, for practical purposes, we should consider portability, flexibility, and power consumption. Therefore, we sought to illustrate that margin-based models are not affected by variations in sensor channels. In this evaluation, we chose different subsets of the 107 sensor channels as our datasets, based on Principal Component Analysis (PCA), from the OPPORTUNITY dataset. By obtaining the top *n* sensor channels according to their variance rank results, we carried out experiments with subsets for n∈{20,50,80}. The other settings were kept the same as in previous experiments.

**Figure 6:**
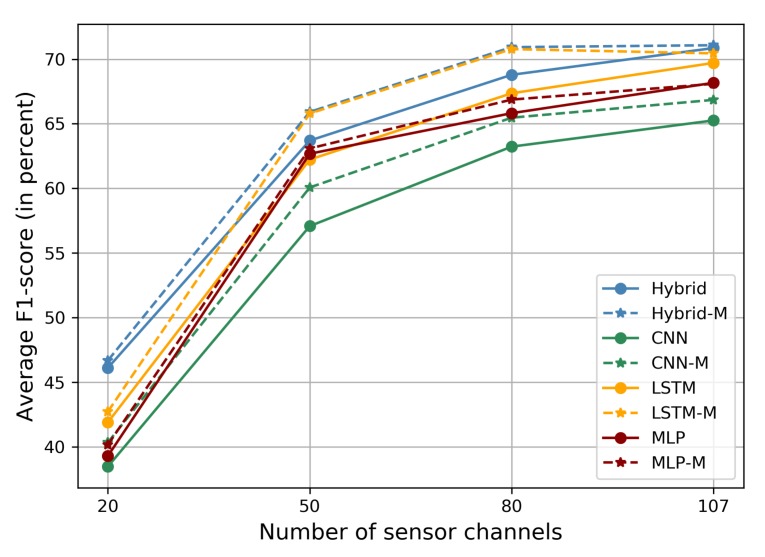
The average F1-score (in percent) using different numbers of sensor channels on the OPPORTUNITY dataset.

The average F1-scores are shown in Table 5 and Figure 6. Comparing the deep learning and margin-based models, the average improvements in average F1-score for n={20,50,80,107} were 1.03, 2.30, 2.21, and 0.61, respectively. This reveals that more rich features can be acquired when the number of sensor channels increases. The margin-based method obviously improved the ability of the networks.

#### 5.2.3. Margin Value

**Figure 7:**
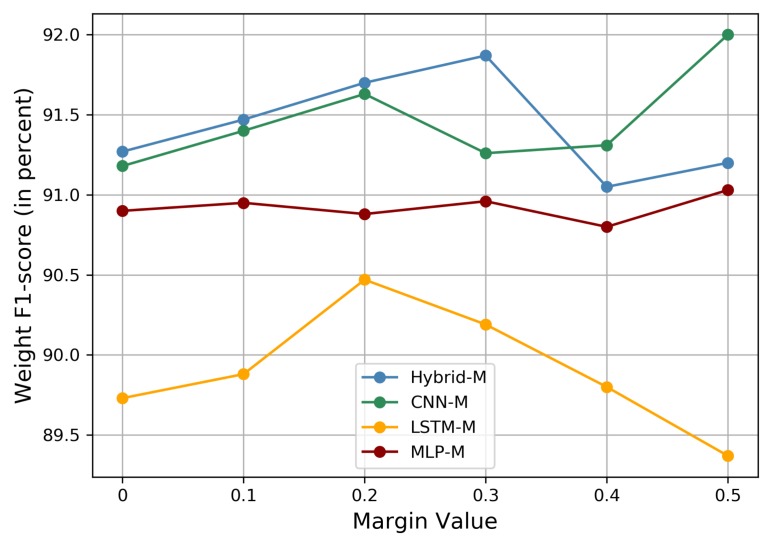
The weighted F1-score (in percent) of the margin-based models for different margin values with the OPPORTUNITY dataset.

Further, the angle interval *m* as a hyper-parameter varied with the network. Therefore, performance plots with different angle interval values on the OPPORTUNITY dataset are shown in Figure 7. The margin value varied for distinct models, which means that a large margin value heavily enhanced intra-class compactness and inter-class diversity uncertainly, which had a large impact on the classification performance of HAR models. The margin values were tailored to the different deep learning models. We set *m* for MLP-M, CNN-M, LSTM-M, and Hybrid-M to 0.5, 0.2, 0.5, and 0.3, respectively, for the OPPORTUNITY dataset. For the UniMiB-SHAR dataset, we used a margin value of 0.1 for CNN-M and left the others unchanged. We set *m* for MLP-M and CNN-M to 0.1 and 0.3, respectively, for the PAMAP2 dataset. As the margin value was increased, the discriminative power of the extracted features could be significantly enhanced.

#### 5.2.4. Comparison with Softmax Loss

**Figure 8:**
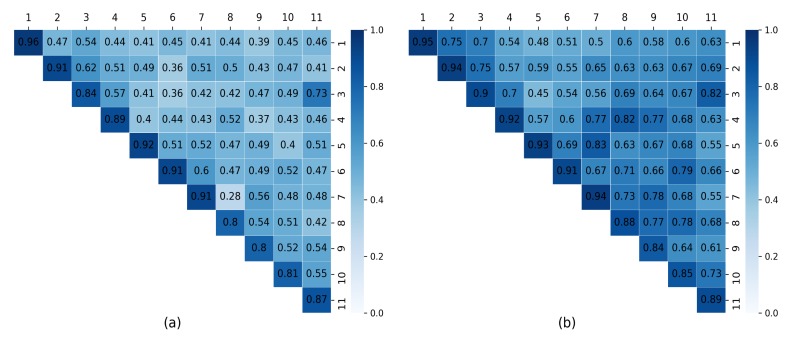
Heatmaps of cosine similarity of all classes using the CNN model on the PAMAP2 testing dataset: (**a**) softmax loss; and (**b**) additive angular margin loss.

To show that margin-based loss can obtain more discriminative features, compared with softmax loss, we carried out an experiment to calculate the cosine similarity (i.e., intra-class and inter-class) on the PAMAP2 testing dataset. Due to its better performance on the PAMAP2 dataset, the CNN-M model was used for this experiment. We extracted features from the first fully-connected layer for margin-based and softmax losses, respectively. Then, we calculated the cosine similarities within and between classes. A heatmap of these similarities is pictured in Figure 8. Compared with the similarities obtained by softmax loss (Figure 8b), the similarities gained through margin-based loss (Figure 8a) were larger intra-class and smaller inter-class. The results of this experiment demonstrate that our margin-based method can improve the discriminative power of networks, which is significant for deep learning networks which attempt to distinguish similar human activities.

**Figure 9:**
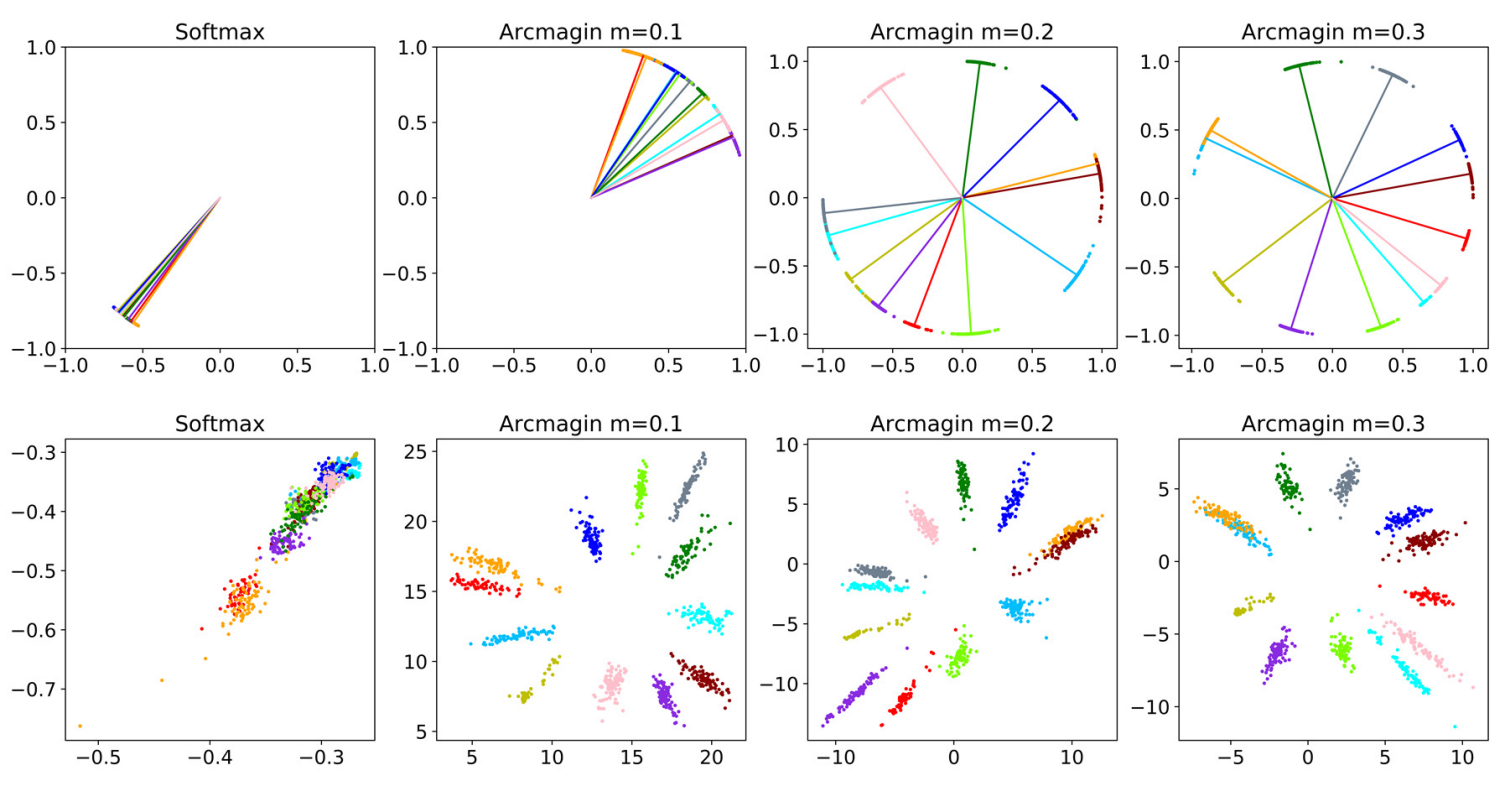
Testing softmax and arcmargin losses on the PAMAP2 validation dataset with 2D features. In this experiment, we used a CNN model to learn 2D features on the validation set of the PAMAP2 dataset. To realise this, we set the output dimension of the first fully-connected layer to 2. The first and second rows are the features in Euclidean space and angular space, respectively. Dots of different colours represent the features of different classes.

To better visualise the features and prove the effectiveness of our approach, we performed a experiment using the CNN-M network on the PAMAP2 validation dataset. We selected data segments from 12 classes, including 1200 samples (100 samples/class), to train 2D feature embedding networks with the softmax loss and margin-based loss with different margin values. In Figure 9, the 2D feature distributions in Euclidean space and angular space are shown in the first and second row, respectively. We can observe that the softmax loss did not provide separable features, while the margin-based loss enforced an evident gap among different classes.

### 5.3. Performance of Open-Set HAR

**Figure 10:**
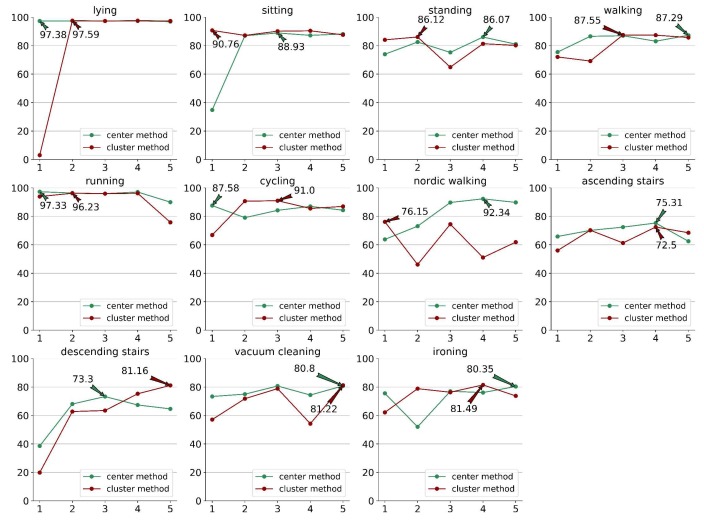
The F1-score (in percent) of each class in the PAMAP2 dataset evaluated by the CNN-M model for open-set human activity recognition. Among, the horizontal axis represents the number of chosen seed. The two lines represent the center and cluster methods. The markpoints of different lines represent the highest F1-score.

We used the PAMAP2 dataset to evaluate open-set HAR performance. For each class of the PAMAP2 dataset, we chose data of the class as a new activity and trained our model using data from only the other classes. Based on the performance of the margin-based models on the PAMAP2 dataset, as shown in Table 2, we selected CNN-M as the base model. Figure 10 shows the F1-score of each new class using the center and cluster methods described in Section 3. The mean F1-score, which calculates the mean value of all new classes of PAMAP2, was 90.92% for the closed-set HAR problem using this procedure. In the case of the open-set HAR problem, the center and cluster methods achieved 86.06% and 85.58% mean F1-scores, respectively. The performance of our CNN-M model with open-set HAR data dropped a little, but it is a more practical method in realistic situations. Further, the F1-scores of the CNN-M model were 97.33% and 96.23%, respectively, for the running class that accounted for only 3.59% of all data considered as a minority class. However, CNN achieved an F1-score of only 96% with closed-set HAR. The results of this experiment demonstrate that our margin-based model is robust in the presence of changing data quantities.

## 6. Conclusions

This paper applied a margin mechanism to deal with two well-known issues in HAR. The first one is intra-class diversity and inter-class similarity. We added the proposed margin mechanism to four different deep learning networks and conducted experiments on three benchmark datasets. The results demonstrated that the proposed method could improve the classification performance of the four networks. The proposed method outperformed three machine learning classifers on the OPPORTUNITY dataset. When the classifiers were trained with features extracted from deep learning networks, their performances were significantly improved. It was revealed that the margin mechanism could obtain discriminative features. We also carried out experiments with various hyperparameters on the OPPORTUNITY dataset, which demonstrated the effectiveness of the proposed method under different settings. Furthermore, we visualised the cosine similarity and 2-D features of the softmax and margin-modified losses. The figures revealed that the margin mechanism could learn more feature representations which have small inter-class scatter but large inter-class separation. Finally, we attempted to solve the second problem: open-set human activity recognition. We conducted experiments on the PAMAP2 dataset with our CNN-M model, and the results showed strong performance when treating each class as a new activity. The results of this experiment illustrate that our approach is, indeed, useful for open-set HAR.

## Figures and Tables

**Table 1:** Settings of the models used on the OPPORTUNITY, UniMiB-SHAR, and PAMAP2 datasets. 1 and 2 indicate the number of LSTM cells used in the OPPORTUNITY and PAMAP2 datasets, respectively.

	OPPORTUNITY${}^{1}$ and PAMAP2${}^{2}$		UniMiB-SHAR	
Model	Parameter	Value	Parameter	Value
MLP	Neurons in fully-connected layers 1, 2, and 3	2000	Neurons in fully-connected layers 1, 2, and 3	6000
CNN	Convolutional kernel size for blocks 1, 2, and 3	(11,1), (10,1), (6,1)	Convolutional kernel size for block 1	(32,3)
	Convolutional sliding stride for blocks 1, 2, and 3	(1,1), (1,1), (1,1)	Convolutional sliding stride for block 1	(1,1)
	Convolutional kernels for blocks 1, 2, and 3	50, 40, 30	Convolutional kernels for block 1	100
	Pooling sizes for blocks 1, 2, and 3	(2,1), (3,1), (1,1)	Pooling sizes for block 1	(2,1)
	Neurons in fully-connected layer	1000	Neurons in fully-connected layer	6000
LSTM	LSTM cells in layers 1 and 2	64${}^{1}$, 64${}^{1}$, 170${}^{2}$, 170${}^{2}$	LSTM cells in layers 1 and 2	151, 151
	Output dimensions of LSTM cells in layers 1 and 2	600, 600	Output dimensions of LSTM cells in layers 1 and 2	1000, 1000
	Neurons in fully-connected layer	512	Neurons in fully-connected layer	6000
Hybrid	Convolutional kernel size for block 1	(11,1)	Convolutional kernel size for block 1	(32,3)
	Convolutional sliding stride for block 1	(1,1)	Convolutional sliding stride for block 1	(1,1)
	Convolutional kernels for block 1	50	Convolutional kernels for block 1	100
	Pooling sizes for block 1	(2,1)	Pooling sizes for block 1	(2,1)
	LSTM cells in layers 1 and 2	27${}^{1}$, 27${}^{1}$, 80${}^{2}$, 80${}^{2}$	LSTM cells in layers 1 and 2	60, 60
	Output dimensions of LSTM cells in layers 1 and 2	600, 600	Output dimensions of LSTM cells in layers 1 and 2	1000, 1000
	Neurons in fully-connected layer	512	Neurons in fully-connected layer	6000

**Table 2:** Classification performance results (in percent) of the various models under the OPPORTUNITY, UniMiB-SHAR, and PAMAP2 datasets. ’-M’ represents models utilising an arcmargin layer.

	OPPORTUNITY			UniMiB-SHAR			PAMAP2		
Method	Acc	$F_{w}$	$F_{m}$	Acc	$F_{w}$	$F_{m}$	Acc	$F_{w}$	$F_{m}$
HC [5]	89.96	89.53	63.76	32.01	22.85	13.78	-	-	-
CBH [5]	89.66	88.99	62.27	75.21	74.13	60.01	-	-	-
CBS [5]	90.22	89.88	67.50	77.03	75.93	63.23	-	-	-
AE [5]	87.80	87.60	55.62	65.67	64.84	55.04	-	-	-
MLP [5]	91.11	90.86	68.17	71.62	70.81	59.97	82.63	80.83	72.92
CNN [5]	90.58	90.19	65.26	74.97	74.29	64.65	91.51	91.35	83.34
LSTM [5]	91.29	91.16	69.71	71.47	70.82	59.32	84.00	82.71	74.00
Hybrid [5]	91.76	91.56	70.86	74.63	73.65	64.47	85.12	83.73	76.10
MLP-M	91.28	91.03	68.09	73.94	73.55	61.59	82.47	82.09	74.43
CNN-M	90.88	90.47	66.85	74.86	74.42	63.30	**93.74**	**93.75**	92.95
LSTM-M	**92.30**	**91.99**	70.45	74.17	72.93	59.43	86.00	84.60	83.75
Hybrid-M	91.92	91.87	**71.08**	**77.88**	**77.29**	**65.31**	93.52	93.52	**93.09**

**Table 3:** Classification performance results (in percent) of three machine learning classifiers on the OPPORTUNITY dataset. ’-DF’ and ’-DF-M’ mean that the features used to train the three classifiers were obtained from the LSTM model and the LSTM-M model, respectively.

Method	Acc	$F_{w}$	$F_{m}$
SVM	89.96	89.53	63.76
Random Forest	89.21	87.08	52.45
Naive Bayes	44.79	52.61	32.81
SVM-DF	91.81	91.62	70.24
Random Forest-DF	91.84	91.63	70.24
Naive Bayes-DF	91.15	91.29	69.03
SVM-DF-M	91.88	91.62	70.43
Random Forest-DF-M	91.93	91.64	70.42
Naive Bayes-DF-M	91.68	91.62	70.08
LSTM-M	**92.30**	**91.99**	**70.45**

**Table 4:** Classification performance results (in percent) using different sliding window lengths on the OPPORTUNITY dataset.

	T = 32			T = 64			T = 96		
Method	Acc	$F_{w}$	$F_{m}$	Acc	$F_{w}$	$F_{m}$	Acc	$F_{w}$	$F_{m}$
MLP [5]	90.79	90.40	66.33	91.11	90.86	68.17	90.94	90.65	66.37
CNN [5]	90.34	89.71	62.10	90.58	90.19	65.26	90.38	89.98	63.38
LSTM [5]	90.88	90.60	67.20	91.29	91.16	69.71	91.33	91.21	68.64
Hybrid [5]	91.10	90.75	67.31	91.76	91.56	70.86	91.44	91.25	69.04
MLP-M	91.13	90.77	66.80	91.28	91.03	68.09	91.34	91.10	67.42
CNN-M	89.97	89.87	64.20	90.88	90.47	66.85	91.47	91.16	67.78
LSTM-M	91.34	91.10	68.52	92.30	91.99	70.45	92.02	91.93	71.72
Hybrid-M	92.06	91.77	71.36	91.92	91.87	71.08	92.45	92.22	71.03

**Table 5:** The average F1-score (in percent) using different numbers of sensor channels on the OPPORTUNITY dataset.

Method	20	50	80	107
MLP [5]	39.29	62.68	65.82	68.17
CNN [5]	38.47	57.08	63.23	65.26
LSTM [5]	41.89	62.23	67.36	69.71
Hybrid [5]	46.11	63.70	68.79	70.86
MLP-M	40.16	63.09	66.87	68.09
CNN-M	40.33	60.07	65.47	66.85
LSTM-M	42.72	65.80	70.78	70.45
Hybrid-M	**46.68**	**65.92**	**70.93**	**71.08**
